# Supplementary material for: Tracing In-Hospital COVID-19 Outcomes: A Multistate Model Exploration (TRACE)
Source: Life (Basel). 2024 Sep 21;14(9):1195. doi: 10.3390/life14091195 (PMC11433282; doi:10.3390/life14091195)
Supplement: Supplementary file 1 [file life-14-01195-s001.zip › Supplementary Figures.pdf]

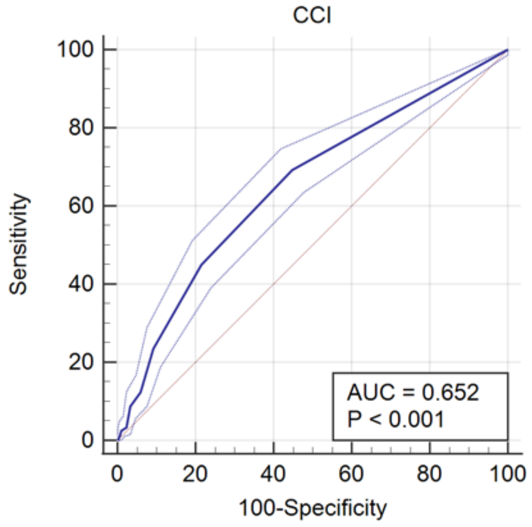

Figure S1. The Receiver Operating Characteristic (ROC) curve illustrates the Charlson Comorbidity Index (CCI) performance for mortality prediction. The plot includes the area under the curve (AUC) to measure the model's discriminative ability. Additionally, the 95% Confidence Interval (CI) bands and the neutral diagonal line are shown, reflecting the precision of the ROC estimates across the curve.

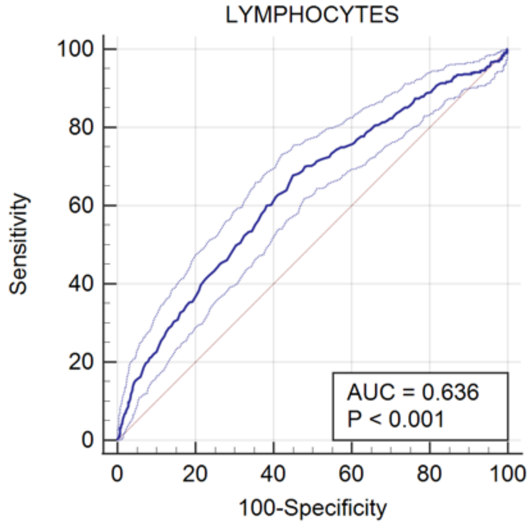

Figure S2. The Receiver Operating Characteristic (ROC) curve illustrates Lymphocyte performance for mortality prediction. The plot includes the area under the curve (AUC) to measure the model's discriminative ability. Additionally, the 95% Confidence Interval (CI) bands and the neutral diagonal line are shown, reflecting the precision of the ROC estimates across the curve.
